# Supplementary material for: Inferring delays in partially observed gene regulation processes
Source: Bioinformatics. 2023 Nov 3;39(11):btad670. doi: 10.1093/bioinformatics/btad670 (PMC10660296; doi:10.1093/bioinformatics/btad670)
Supplement: btad670_Supplementary_Data [file btad670_supplementary_data.pdf]

Inferring delays in partially observed gene regulation processes

Hyukpyo Hong, Mark Jayson Cortez, Yu-Yu Cheng, Hang Joon Kim, Boseung Choi, Krešimir Josić and Jae Kyoung Kim

Supplementary Methods

Role of the indicator function $\chi(\cdot)$ in the approximate likelihood function

Consider a biochemical reaction network with u species $Z_1, \ldots, Z_u$ and v reactions $R_1, \ldots, R_v$ where the reaction R_k is represented as

$$R_k : p_{k1}Z_1 + \cdots + p_{ku}Z_u \to q_{k1}Z_1 + \cdots + q_{ku}Z_u$$

with the stoichiometric coefficients of species Z_j, p_{kj} and q_{kj}.

As shown by Choi *et al.* (2020), the approximate likelihood function is given by

$$\hat{L}\left(\mathbf{r}, \theta, \mathbf{\Delta} | \mathbf{z}\right) = \left[\prod_{i=1}^{T}\prod_{k=1}^{v} \frac{\hat{f}_k(i, \mathbf{z}, \theta_k, \Delta_k)^{r_{ki}}}{r_{ki}!}\right] \times \exp\left(-\hat{\Lambda}_0\left(T, \mathbf{z}, \theta, \mathbf{\Delta}\right)\right) \times \chi(\mathbf{z}|\mathbf{r}), \tag{S1}$$

where

$$\Lambda_0\left(t, \mathbf{z_c}, \theta, \mathbf{\Delta}\right) = \sum_{k=1}^{v} \Lambda_k\left(t, \mathbf{z_c}, \theta_k, \Delta_k\right) \text{ and}$$
$$\Lambda_k\left(t, \mathbf{z_c}, \theta_k, \Delta_k\right) = \int_0^t \hat{f}_k\left(\hat{t}, \mathbf{z_c}, \theta_k, \Delta_k\right) d\hat{t}.$$

Here, $\hat{f}_k$ is the reaction completion propensity of the k-th reaction, and $\mathbf{z}$ is the collection of vectors for the number of species, i.e., the protein counts, at the discrete measurement time points. The collection of parameters $\theta = \{\theta_k\}$ and $\mathbf{\Delta} = \{\Delta_k\}$ define the reaction initiation propensities and delay distributions, respectively, and $\mathbf{r} = (\mathbf{r}_1, \ldots, \mathbf{r}_v)$ is the collection of vectors for reaction counts completing within each of the time intervals, i.e., $\mathbf{r}_k = (r_{k1}, \ldots, r_{kT})$, and r_{ki} is the count of reaction of type k that completes within the time interval $(i-1, i]$.

The indicator function, $\chi(\mathbf{z}|\mathbf{r})$, has value one if the trajectory of species matches the counts of completed reactions and zero otherwise. For example, for a simple birth-death process $\emptyset \to Z \to \emptyset$ with the initial condition $Z(0) = 0$, let $\mathbf{r}_1$ and $\mathbf{r}_2$ be the number of birth (i.e., $\emptyset \to Z$) and death (i.e., $Z \to \emptyset$) reactions, respectively, and suppose that we measured the time series of Z at time $t = 0, 1, 2,$ and 3. If $\mathbf{r}_1 = (3, 2, 4)$ and $\mathbf{r}_2 = (0, 1, 2)$, then $\mathbf{z} = (0, 3, 4, 6)$. The vector $\mathbf{z}$ is unique for a given $\mathbf{r}$, and thus $\chi(\mathbf{z}|\mathbf{r})$ equals one only for this specific $\mathbf{z}$, and equals zero for all other trajectories.

Derivation of the Metropolis-Hastings acceptance probability for reaction counts in the simulation-based MCMC method

In the conventional Metropolis-Hastings algorithm, the acceptance probability for a proposed state x^*, $\rho(x^*, x)$, is given as follows:

$$\rho(x^*, x) = \min\left\{\frac{g(x^*)}{g(x)} \times \frac{q(x|x^*)}{q(x^*|x)}, 1\right\} \tag{S2}$$

where $g(x)$ is a target distribution that one intends to sample from and $q(x^*|x)$ is a proposal distribution for given x.

In our simulation-based MCMC method, the target distribution is the conditional posterior distribution of reaction counts, $\pi(\mathbf{r}|\mathbf{y}_{\text{obs}}, \theta^{(j)}, \mathbf{\Delta}^{(j)}, \sigma)$. The proposal distribution is determined by the stochastic simulation, τ-leaping method, and it is proportional to the approximate likelihood function in Eq. (S1), $\hat{L}\left(\mathbf{r}, \theta^{(j)}, \mathbf{\Delta}^{(j)}|\mathbf{z}\right)$ $= \hat{L}\left(\mathbf{r}, \theta^{(j)}, \mathbf{\Delta}^{(j)}|\bar{\mathbf{x}}(\mathbf{r}), \bar{\mathbf{y}}(\mathbf{r})\right)$ where $\bar{\mathbf{x}}(\mathbf{r})$ and $\bar{\mathbf{y}}(\mathbf{r})$ are the trajectories of unobserved and observed species corresponding to the reaction counts $\mathbf{r}$, respectively. Therefore, we can rewrite the acceptance probability in Eq. (S2) as

$$\begin{aligned}\frac{g(x^*)}{g(x)} \times \frac{q(x|x^*)}{q(x^*|x)} &= \frac{\pi(\mathbf{r}^*|\mathbf{y}_{\text{obs}}, \theta^{(j)}, \mathbf{\Delta}^{(j)}, \sigma)}{\pi\left(\mathbf{r}^{(j)}|\mathbf{y}_{\text{obs}}, \theta^{(j)}, \mathbf{\Delta}^{(j)}, \sigma\right)} \times \frac{\hat{L}\left(\mathbf{r}^{(j)}, \theta^{(j)}, \mathbf{\Delta}^{(j)}|\bar{\mathbf{x}}(\mathbf{r}^{(j)}), \bar{\mathbf{y}}(\mathbf{r}^{(j)})\right)}{\hat{L}\left(\mathbf{r}^*, \theta^{(j)}, \mathbf{\Delta}^{(j)}|\bar{\mathbf{x}}(\mathbf{r}^*), \bar{\mathbf{y}}(\mathbf{r}^*)\right)} \\ &= \frac{\pi(\mathbf{r}^*)\hat{L}(\mathbf{r}^*, \theta^{(j)}, \mathbf{\Delta}^{(j)}|\mathbf{y}_{\text{obs}}, \sigma)}{\pi(\mathbf{r}^{(j)})\hat{L}(\mathbf{r}^{(j)}, \theta^{(j)}, \mathbf{\Delta}^{(j)}|\mathbf{y}_{\text{obs}}, \sigma)} \times \frac{\hat{L}\left(\mathbf{r}^{(j)}, \theta^{(j)}, \mathbf{\Delta}^{(j)}|\bar{\mathbf{x}}(\mathbf{r}^{(j)}), \bar{\mathbf{y}}(\mathbf{r}^{(j)})\right)}{\hat{L}\left(\mathbf{r}^*, \theta^{(j)}, \mathbf{\Delta}^{(j)}|\bar{\mathbf{x}}(\mathbf{r}^*), \bar{\mathbf{y}}(\mathbf{r}^*)\right)}. \qquad \text{(S3)}\end{aligned}$$

Using Eq. (9) in the main text, we can replace the function $\hat{L}(\mathbf{r}, \theta^{(j)}, \mathbf{\Delta}^{(j)}|\mathbf{y}_{\text{obs}}, \sigma)$ in Eq. (S3) with $L(\bar{\mathbf{y}}(\mathbf{r})|\mathbf{y}_{\text{obs}}, \sigma) \times \hat{L}(\mathbf{r}, \theta^{(j)}, \mathbf{\Delta}^{(j)}|\bar{\mathbf{x}}(\mathbf{r}), \bar{\mathbf{y}}(\mathbf{r}))$. We then can re-express Eq. (S3) as follows:

$$\frac{\pi(\mathbf{r}^*)L(\bar{\mathbf{y}}(\mathbf{r}^*)|\mathbf{y}_{\text{obs}}, \sigma)\hat{L}(\mathbf{r}^*, \theta^{(j)}, \mathbf{\Delta}^{(j)}|\bar{\mathbf{x}}(\mathbf{r}^*), \bar{\mathbf{y}}(\mathbf{r}^*))}{\pi(\mathbf{r}^{(j)})L(\bar{\mathbf{y}}(\mathbf{r}^{(j)})|\mathbf{y}_{\text{obs}}, \sigma)\hat{L}(\mathbf{r}^{(j)}, \theta^{(j)}, \mathbf{\Delta}^{(j)}|\bar{\mathbf{x}}(\mathbf{r}^{(j)}), \bar{\mathbf{y}}(\mathbf{r}^{(j)}))} \times \frac{\hat{L}\left(\mathbf{r}^{(j)}, \theta^{(j)}, \mathbf{\Delta}^{(j)}|\bar{\mathbf{x}}(\mathbf{r}^{(j)}), \bar{\mathbf{y}}(\mathbf{r}^{(j)})\right)}{\hat{L}\left(\mathbf{r}^*, \theta^{(j)}, \mathbf{\Delta}^{(j)}|\bar{\mathbf{x}}(\mathbf{r}^*), \bar{\mathbf{y}}(\mathbf{r}^*)\right)}.$$

After canceling common terms, we obtain Eq. (10) in the main text:

$$\frac{\pi(\mathbf{r}^*)L(\bar{\mathbf{y}}(\mathbf{r}^*)|\mathbf{y}_{\text{obs}}, \sigma)}{\pi(\mathbf{r}^{(j)})L(\bar{\mathbf{y}}(\mathbf{r}^{(j)})|\mathbf{y}_{\text{obs}}, \sigma)} = \frac{\pi(\mathbf{r}^*)L(\mathbf{y}^*|\mathbf{y}_{\text{obs}}, \sigma)}{\pi(\mathbf{r}^{(j)})L(\mathbf{y}^{(j)}|\mathbf{y}_{\text{obs}}, \sigma)}$$

Explicit formula of the approximate likelihood for the two-step activation model

In the following, we use the notation introduced in the main text. From Eq. (9) in the main text, we have

$$\hat{L}(\mathbf{r}, \theta, \mathbf{\Delta}|\mathbf{y}_{\text{obs}}, \sigma) = L(\bar{\mathbf{y}}(\mathbf{r})|\mathbf{y}_{\text{obs}}, \sigma) \times \hat{L}\left(\mathbf{r}, \theta, \mathbf{\Delta}|\bar{\mathbf{x}}(\mathbf{r}), \bar{\mathbf{y}}(\mathbf{r})\right). \qquad \text{(S4)}$$

For the two-step activation model, the unobserved and observed species are denoted by X and Y, respectively, and the reaction counts are denoted by $\mathbf{r} = (\mathbf{r}_1, \mathbf{r}_2, \mathbf{r}_3, \mathbf{r}_4)$ where $\mathbf{r}_1, \mathbf{r}_2, \mathbf{r}_3$, and $\mathbf{r}_4$ are the vectors of counts of the production of X, the decay of X, the production of Y, and the decay of Y, respectively, completing within each of the time intervals. The vector of the kinetic parameters, θ, is given by (A_X, K_M, A_Y, B), and the delay parameters $\mathbf{\Delta}$ is given by $(\alpha_X, \beta_X, \alpha_Y, \beta_Y)$. For notational simplicity, we let $\bar{X}_r(i)$, $\bar{Y}_r(i)$, and $Y_{\text{obs}}(i)$ be the values of $\bar{\mathbf{x}}(\mathbf{r})$, $\bar{\mathbf{y}}(\mathbf{r})$, and $\mathbf{y}_{\text{obs}}$ evaluated at time i, respectively.

Since the observational noise, $\epsilon(t)$, is i.i.d. and follows $N(0, \sigma^2)$, the likelihood function for the observed processes, $L(\mathbf{y}_{\text{obs}}|\bar{\mathbf{y}}(\mathbf{r}))$ is given by $\prod_{i=0}^{T} p(Y_{\text{obs}}(i) - \bar{Y}_r(i); 0, \sigma^2)$ where $p(\cdot; \mu, \sigma^2)$ is the probability density function of the normal distribution with the mean μ and the variance σ^2. Then Eq. (S4) can be re-expressed as follows:

$$\hat{L}(\mathbf{r}, \theta, \mathbf{\Delta}|\mathbf{y}_{\text{obs}}, \sigma) = \prod_{i=0}^{T} p(Y_{\text{obs}}(i) - \bar{Y}_r(i); 0, \sigma^2) \times \left[\prod_{i=1}^{T}\prod_{k=1}^{4} \frac{\hat{f}_k(i, \mathbf{z}, \theta_k, \Delta_k)^{r_{ki}}}{r_{ki}!}\right] \exp\left(-\hat{\Lambda}_0\left(T, \mathbf{z}, \theta, \mathbf{\Delta}\right)\right) \times \chi(\mathbf{z}|\mathbf{r})$$

where $\hat{f}_k$ is the approximate completion propensity for the k-th reaction given as follows:

$$\begin{aligned}\hat{f}_1(i, \mathbf{z}, \theta_1, \Delta_1) &= A_X \int_{i-1}^{i} Ga(t; \alpha_X, \beta_X)dt, \\ \hat{f}_2(i, \mathbf{z}, \theta_2, \Delta_2) &= B\left(\frac{\bar{X}_r(i-1) + \bar{X}_r(i)}{2}\right), \\ \hat{f}_3(i, \mathbf{z}, \theta_3, \Delta_3) &= A_Y \sum_{m=0}^{i-1}\left[\frac{\bar{X}_r(i-1-m)}{K_M + \bar{X}_r(i-1-m)} \times C(m) + \frac{\bar{X}_r(i-m)}{K_M + \bar{X}_r(i-m)} \times D(m)\right], \qquad \text{(S5)} \\ \hat{f}_4(i, \mathbf{z}, \theta_4, \Delta_4) &= B\left(\frac{\bar{Y}_r(i-1) + \bar{Y}_r(i)}{2}\right).\end{aligned}$$

Here, $Ga(t; \alpha_X, \beta_X)$ is the cumulative density function of the gamma distribution with the shape parameter α_X and the rate parameter β_X, so $A_X \int_{i-1}^{i} Ga(t; \alpha_X, \beta_X)dt$ represents an activation rate reflecting the delay compared with the full activation, A_X. Note that if there are multiple observed trajectories, we can regard them as independent, so the product of the likelihood function for each trajectory can be the likelihood for the given multiple trajectories.

To compute $C(m)$ and $D(m)$, let us recall the reaction completion propensity formula Eq. (6) for the production of Y:

$$\hat{f}_3(i, \mathbf{z}, (A_Y, K_M), (\alpha_Y, \beta_Y)) = \sum_{m=0}^{i-1} \int_m^{m+1} \int_{t-1}^{t} \left[(s+1-t)\, A_Y \frac{\bar{X}_r(i-1-m)}{K_M + \bar{X}_r(i-1-m)} + (t-s) A_Y \frac{\bar{X}_r(i-m)}{K_M + \bar{X}_r(i-m)} \right] dga(s; \alpha_Y, \beta_Y)dt$$

where $ga(s; \alpha_Y, \beta_Y)$ is a probability density function of the gamma distribution with the shape parameter α_Y and the rate parameter β_Y. By simplifying this equation, we obtain Eq. (S5) with $C(m)$ and $D(m)$ given by

$$C(m) = \int_m^{m+1} \left[(1-t) \int_{t-1}^{t} ga(s; \alpha_Y, \beta_Y)ds + \frac{\alpha_Y}{\beta_Y} \int_{t-1}^{t} ga(s; \alpha_Y + 1, \beta_Y)ds] \right] dt,$$

$$D(m) = \int_m^{m+1} \left[t \int_{t-1}^{t} ga(s; \alpha_Y, \beta_Y)ds - \frac{\alpha_Y}{\beta_Y} \int_{t-1}^{t} ga(s; \alpha_Y + 1, \beta_Y)ds] \right] dt.$$

When we estimate the kinetic and delay parameters, we fix the parameter for the observational noise, σ. To obtain estimates from simulated data (Figs. 3 and 4), we set $\sigma = \sigma_e = 10$. The generative model and the inference model are thus mismatched because the noise in the generative model, proportional to the state of $Y(t)$, i.e., $N(0, Y(t))$, is not taken into account in the inference model. We made this choice intentionally to mimic the real experiment (Fig. 5) where it is hard to quantify the exact noise structure. Therefore, we fix σ for inference using experimental data as the basal noise, which is separately estimated using the observed time series before the introduction of the inducer, IPTG (i.e., before t=0). Specifically, we use standard deviation of time series before $t = 0$ as the estimated σ value.

Description of the simulation-based Bayesian MCMC method for a two-step activation model

We illustrate our simulation-based Bayesian MCMC method using a stochastic two-step activation model (Fig. 3a). This method is basically a Metropolis-Hastings (MH) within Gibbs sampling approach. However, the difference is that we use a stochastic simulation to generate proposal reaction counts $\mathbf{r}$ while we use direct sampling or the MH algorithm to sample the other parameters.

The posterior distribution of the parameters for measured trajectories $\mathbf{y}_{\text{obs}}(t)$ at discrete time points, $t = 0, \ldots, T$, is given by

$$\pi(\mathbf{r}, A_X, A_Y, K_M, B, \alpha_X, \beta_X, \alpha_Y, \beta_Y | \mathbf{y}_{\text{obs}}, \sigma) \propto \pi(A_X)\pi(A_Y)\pi(K_M)\pi(B)\pi(\alpha_X)\pi(\beta_X)\pi(\alpha_Y)\pi(\beta_Y)\hat{L}(\mathbf{r}, A_X, A_Y, K_M, B, \alpha_X, \beta_X, \alpha_Y, \beta_Y | \mathbf{y}_{\text{obs}}, \sigma),$$

where $\pi(A_X), \ldots, \pi(\beta_Y)$ are the prior distributions, and $\hat{L}$ is the approximate likelihood function. For all the parameter inferences, we used non-informative gamma priors, $\Gamma(10^{-6}, 10^{-6})$. Because the likelihood functions for A_X, A_Y, and B are proportional to gamma distributions, we can directly sample them from

each of the conditional posteriors given on the other parameters. Specifically,

$$
\begin{aligned}
&A_X|\mathbf{y}_{\text{obs}},\mathbf{r},A_Y,K_M,B,\alpha_X,\beta_X,\alpha_Y,\beta_Y,\sigma \\
&\qquad\sim\Gamma\left(\sum_{i=1}^{T} r_{1i}+10^{-6},\sum_{i=1}^{T}\int_{i-1}^{i} Ga(t;\alpha_X,\beta_X)dt+10^{-6}\right). \\
&A_Y|\mathbf{y}_{\text{obs}},\mathbf{r},A_X,K_M,B,\alpha_X,\beta_X,\alpha_Y,\beta_Y,\sigma \\
&\qquad\sim\Gamma\left(\sum_{i=1}^{T} r_{3i}+10^{-6},\sum_{i=1}^{T}\left[\frac{\bar{X}_r(i-m)}{K_M+\bar{X}_r(i-m)}\times C(m)+\frac{\bar{X}_r(i-m+1)}{K_M+\bar{X}_r(i-m+1)}\times D(m)\right]+10^{-6}\right). \\
&B|\mathbf{y}_{\text{obs}},\mathbf{r},A_X,A_Y,K_M,\alpha_X,\beta_X,\alpha_Y,\beta_Y,\sigma \\
&\qquad\sim\Gamma\left(\sum_{i=1}^{T} r_{2i}+r_{4i}+10^{-6},\sum_{i=1}^{T}\frac{\bar{X}_r(i-1)+\bar{X}_r(i)+\bar{Y}_r(i-1)+\bar{Y}_r(i)}{2}+10^{-6}\right).
\end{aligned}
\tag{S6}
$$

For the rest parameters, $K_M, \alpha_X, \beta_X, \alpha_Y, \beta_Y$, their conditional posterior distributions are not well-known distributions, so we use the Robust Adaptive Metropolis algorithm (Vihola, 2012) to sample from their conditional posterior distributions. For reaction counts $\mathbf{r}$, we use a stochastic simulation, τ-leaping method (Gillespie, 2001).

In sum, our simulation-based Bayesian MCMC for the two-step activation model is described by the following steps.

Step 1. Initialize the kinetic and delay parameters, $A_X^{(0)}, A_Y^{(0)}, K_M^{(0)}, B^{(0)}, \alpha_X^{(0)}, \beta_X^{(0)}, \alpha_Y^{(0)}$,and $\beta_Y^{(0)}$; and the reaction counts $\mathbf{r}^{(0)}$ using the initial parameters and the stochastic simulation.

Step 2. Using the parameter set at the j-th iteration, we generate the proposal reaction counts $\mathbf{r}^*$, which automatically determines corresponding trajectories $\bar{X}_r^*$ and $\bar{Y}_r^*$. We then accept the proposal reaction counts based on the acceptance probability derived in the previous section (Eq. (S2))

Step 3. Draw posterior samples for A_X, A_Y, B from their conditional gamma posterior distributions given by Eq. (S6). For the rest parameters, $K_M, \alpha_X, \beta_X, \alpha_Y, \beta_Y$, we use the Robust Adaptive Metropolis algorithm (Vihola, 2012) to draw from their conditional posterior samples.

Step 4. Repeat Steps 2–3 until a convergence criterion is met.

We typically used 110,000 iterations of Steps 2–4. The first 10,000 iterations were discarded as a burn-in period to avoid the initial condition dependency. The last 100,000 iterations were thinned by a factor of 100 to obtain independent samples from a posterior distribution. Thus, the remaining 1,000 samples were saved to estimate the parameters. Note that if there are multiple observed trajectories, we propose reaction counts for each trajectory and accept or reject them individually.

Explicit formula of the approximate likelihood for a more complex model (Fig. S5)

From Eq. (9) in the main text, we have

$$\hat{L}(\mathbf{r},\theta,\mathbf{\Delta}|\mathbf{y}_{\text{obs}},\sigma)=L(\bar{\mathbf{y}}(\mathbf{r})|\mathbf{y}_{\text{obs}},\sigma)\times\hat{L}\left(\mathbf{r},\theta,\mathbf{\Delta}|\bar{\mathbf{x}}(\mathbf{r}),\bar{\mathbf{y}}(\mathbf{r})\right). \tag{S7}$$

Based on Eq. (S7), we derive the approximate likelihood for a model describing a network of three genes (Fig. **S5**). We assume that we can observe two types of proteins, Y_1 and Y_2, and we cannot observe protein X. Each protein promotes another gene's expression.

The reaction counts are denoted by $\mathbf{r}=(\mathbf{r}_1,\mathbf{r}_2,\mathbf{r}_3,\mathbf{r}_4,\mathbf{r}_5,\mathbf{r}_6)$ where $\mathbf{r}_1,\mathbf{r}_3,\mathbf{r}_5$, $\mathbf{r}_2$, $\mathbf{r}_4$, and $\mathbf{r}_6$ are the vectors of counts of the productions of X, Y_1, Y_2 and the decays of X, Y_1, Y_2, respectively, completing within each of the time intervals. The vector of the kinetic parameters, θ, is given by $(A_X, A_{Y_1}, A_{Y_2}, A_{Y_2\to X}, B, K_{M_1}, K_{M_2}, K_{M_3})$, and the delay parameters $\mathbf{\Delta}$ is given by $(\alpha_X,\beta_X,\alpha_{Y_1},\beta_{Y_1},\alpha_{Y_2},\beta_{Y_2})$. We let $\bar{X}_r(i)$, $\bar{Y}_{1r}(i)$, $\bar{Y}_{2r}(i)$, $Y_{1,\text{ obs}}(i)$, and $Y_{2,\text{ obs}}(i)$ be the values of $\bar{\mathbf{x}}(\mathbf{r})$, $\bar{\mathbf{y}}_\mathbf{1}(\mathbf{r})$, $\bar{\mathbf{y}}_\mathbf{2}(\mathbf{r})$, $\mathbf{y}_{1,\text{obs}}$, and $\mathbf{y}_{2,\text{obs}}$ evaluated at time i, respectively.

Since the observational noise, $\epsilon(t)$, is i.i.d. and follows $N(0,\sigma^2)$, the likelihood function for the observed processes, $L(\bar{\mathbf{y}}_1(\mathbf{r})|\mathbf{y}_{l,\text{obs}},\sigma)$ is given by $\prod_{i=0}^{T} p(Y_{l,\text{obs}}(i) - \bar{Y}_{lr}(i); 0, \sigma^2)$ for $l = 1, 2$ where $p(\cdot;\mu,\sigma^2)$ is the probability density function of the normal distribution with the mean μ and the variance σ^2. Then Eq. (S7) can be re-expressed as

$$\hat{L}(\mathbf{r},\theta,\mathbf{\Delta}|\mathbf{y}_{1,\text{obs}},\mathbf{y}_{2,\text{obs}},\sigma) = \prod_{i=0}^{T}\prod_{l=1}^{2} p(Y_{l,\text{obs}}(i) - \bar{Y}_{lr}(i); 0, \sigma^2)$$
$$\times \left[\prod_{i=1}^{T}\prod_{k=1}^{6} \frac{\hat{f}_k(i,\mathbf{z},\theta_k,\Delta_k)^{r_{ki}}}{r_{ki}!}\right] \exp\left(-\hat{\Lambda}_0\left(T,\mathbf{z},\theta,\mathbf{\Delta}\right)\right) \times \chi(\mathbf{z}|\mathbf{r})$$

where $\hat{f}_k$ is the approximate completion propensity for the k-th reaction given as follows:

$$\hat{f}_1(i,\mathbf{z},\theta_1,\Delta_1) = A_X \int_{i-1}^{i} Ga(t;\alpha_X,\beta_X)dt$$
$$+ A_{Y_2\to X} \sum_{m=0}^{i-1}\left[\frac{\bar{Y}_{2r}(i-1-m)}{K_{M_3}+\bar{Y}_{2r}(i-1-m)} \times C_3(m) + \frac{\bar{Y}_{2r}(i-m)}{K_{M_3}+\bar{Y}_{2r}(i-m)} \times D_3(m)\right], \quad \text{(S8)}$$
$$\hat{f}_2(i,\mathbf{z},\theta_2,\Delta_2) = B\left(\frac{\bar{X}_r(i-1)+\bar{X}_r(i)}{2}\right),$$
$$\hat{f}_3(i,\mathbf{z},\theta_3,\Delta_3) = A_{Y_1} \sum_{m=0}^{i-1}\left[\frac{\bar{X}_r(i-1-m)}{K_{M_1}+\bar{X}_r(i-1-m)} \times C_1(m) + \frac{\bar{X}_r(i-m)}{K_{M_1}+\bar{X}_r(i-m)} \times D_1(m)\right], \quad \text{(S9)}$$
$$\hat{f}_4(i,\mathbf{z},\theta_4,\Delta_4) = B\left(\frac{\bar{Y}_{1r}(i-1)+\bar{Y}_{1r}(i)}{2}\right),$$
$$\hat{f}_5(i,\mathbf{z},\theta_5,\Delta_5) = A_{Y_2} \sum_{m=0}^{i-1}\left[\frac{\bar{Y}_{1r}(i-1-m)}{K_{M_2}+\bar{Y}_{1r}(i-1-m)} \times C_2(m) + \frac{\bar{Y}_{1r}(i-m)}{K_{M_2}+\bar{Y}_{1r}(i-m)} \times D_2(m)\right], \quad \text{(S10)}$$
$$\hat{f}_6(i,\mathbf{z},\theta_6,\Delta_6) = B\left(\frac{\bar{Y}_{2r}(i-1)+\bar{Y}_{2r}(i)}{2}\right).$$

We can compute $C_k(m)$ and $D_k(m)$ for $k = 1, 2, 3$ using the same procedure as when computing $C(m)$ and $D(m)$ in Eq. (S5). These are given by

$$C_1(m) = \int_{m}^{m+1}\left[(1-t)\int_{t-1}^{t} ga(s;\alpha_{Y_1},\beta_{Y_1})ds + \frac{\alpha_{Y_1}}{\beta_{Y_1}}\int_{t-1}^{t} ga(s;\alpha_{Y_1}+1,\beta_{Y_1})ds\right] dt,$$
$$D_1(m) = \int_{m}^{m+1}\left[t\int_{t-1}^{t} ga(s;\alpha_{Y_1},\beta_{Y_1})ds - \frac{\alpha_{Y_1}}{\beta_{Y_1}}\int_{t-1}^{t} ga(s;\alpha_{Y_1}+1,\beta_{Y_1})ds\right] dt,$$
$$C_2(m) = \int_{m}^{m+1}\left[(1-t)\int_{t-1}^{t} ga(s;\alpha_{Y_2},\beta_{Y_2})ds + \frac{\alpha_{Y_2}}{\beta_{Y_2}}\int_{t-1}^{t} ga(s;\alpha_{Y_2}+1,\beta_{Y_2})ds\right] dt,$$
$$D_2(m) = \int_{m}^{m+1}\left[t\int_{t-1}^{t} ga(s;\alpha_{Y_2},\beta_{Y_2})ds - \frac{\alpha_{Y_2}}{\beta_{Y_2}}\int_{t-1}^{t} ga(s;\alpha_{Y_2}+1,\beta_{Y_2})ds\right] dt,$$
$$C_3(m) = \int_{m}^{m+1}\left[(1-t)\int_{t-1}^{t} ga(s;\alpha_X,\beta_X)ds + \frac{\alpha_X}{\beta_X}\int_{t-1}^{t} ga(s;\alpha_X+1,\beta_X)ds\right] dt,$$
$$D_3(m) = \int_{m}^{m+1}\left[t\int_{t-1}^{t} ga(s;\alpha_X,\beta_X)ds - \frac{\alpha_X}{\beta_X}\int_{t-1}^{t} ga(s;\alpha_X+1,\beta_X)ds\right] dt.$$

Supplementary Figures

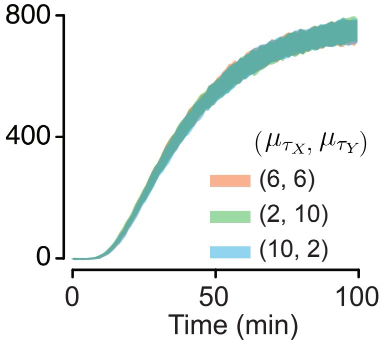

Fig. S1: The simulated measurements of $Y_{\text{obs}}(t)$ are indistinguishable when varying μ_{τ_X} and μ_{τ_Y} while keeping their sum constant. The upper and lower boundaries of the shaded region correspond to the mean±SD for the 40 trajectories obtained for each parameter set. The parameters, $(\alpha_X, \beta_X, \alpha_Y, \beta_Y)$, used for the red, green, and blue regions are $(3.6, 0.6, 3.6, 0.6)$, $(1.2, 0.6, 6.0, 0.6)$, and $(6.0, 0.6, 1.2, 0.6)$, respectively. The kinetic parameters used for these data are the same to those used for the data in Fig. 3b

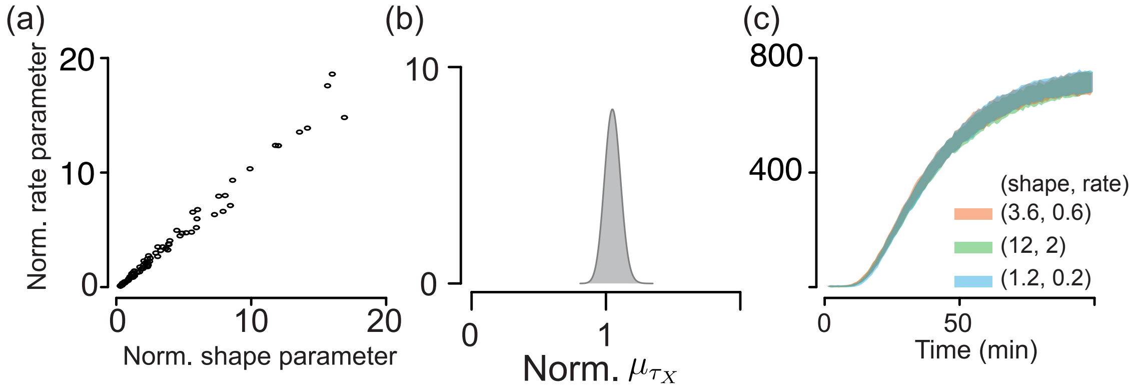

Fig. S2: Unidentifiability of the shape and rate parameters for a Gamma-distributed delay. (**a**) Estimated shape and rate parameters for the Gamma-distributed delay, τ_X, from the analysis in Fig. 3. The posterior distribution shows that the estimates of these parameters are strongly correlated. (**b**) Estimated mean delay. (**c**) The simulated measurements of $Y_{\text{obs}}(t)$ are indistinguishable when varying the shape and rate parameters while keeping their ratio constant. The upper and lower boundaries of the shaded region correspond to the mean±SD for the 40 trajectories obtained for each parameter set.

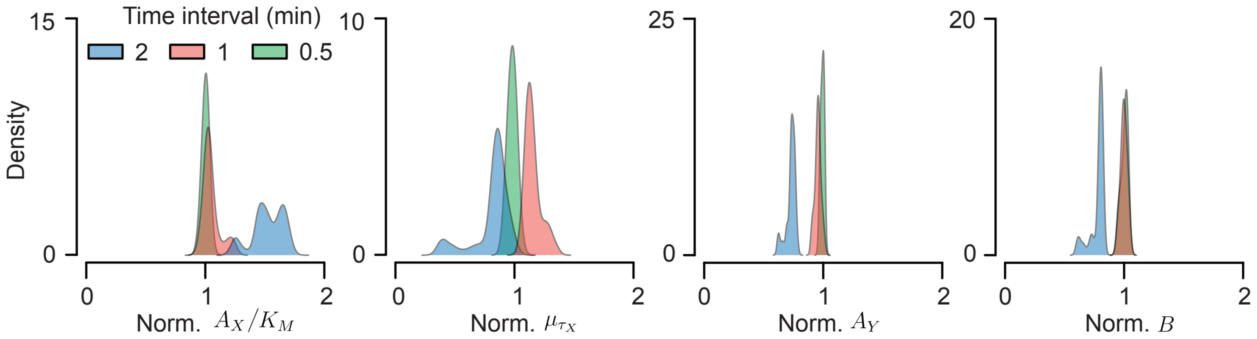

Fig. S3: Estimation results of parameters A_X/K_M, A_Y, τ_X, and B using the 40 trajectories in Fig. 3b in the main text. The estimates became more accurate and precise when the time interval between measurements was shortened.

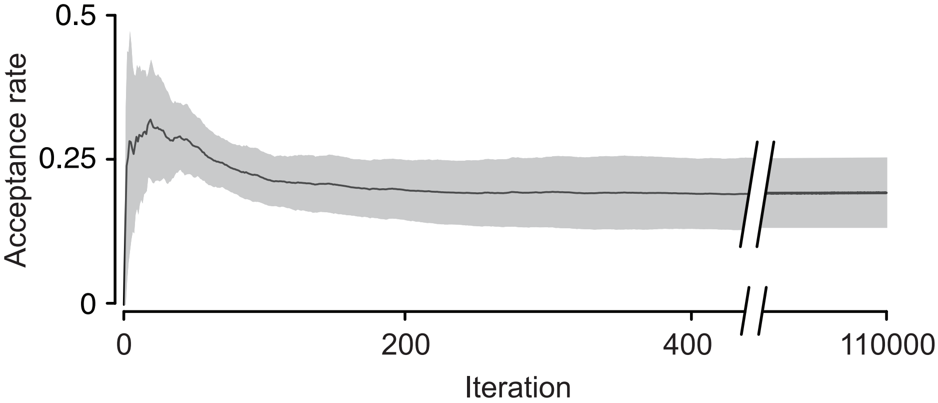

Fig. S4: The acceptance rate of reaction counts for 40 trajectories in Fig. 4a. The black solid line indicates the mean acceptance rate, and the lower and upper boundaries of the shaded region indicate the mean±standard deviation of the acceptance rates.

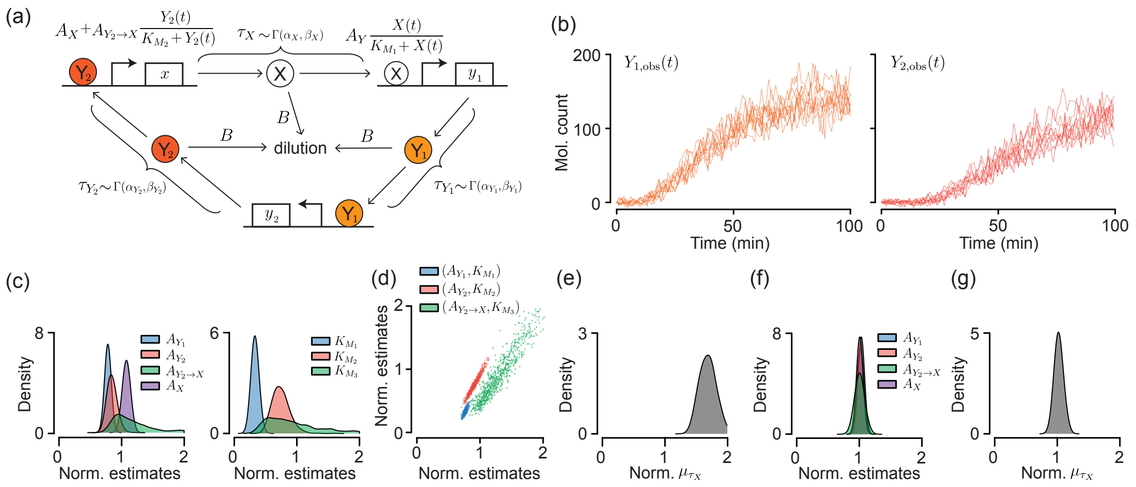

Fig. S5: Illustration of the scalability of our method using a regulatory network with three genes. (**a**) Diagram of the gene regulatory network. We assume that while product X of gene x is unobserved, the counts of proteins Y_1 and Y_2 are observed. (**b**) Ten trajectories of measurements, $Y_{1,\text{ obs}}(t)$ and $Y_{2,\text{ obs}}(t)$ with kinetic parameters $A_X = A_{Y_1} = A_{Y_2} = A_{Y_2 \to X} = 10\text{min}^{-1}$, $B = 0.05\text{min}^{-1}$, $K_{M_1} = K_{M_2} = K_{M_3} = 100$, and delay $\tau_X \sim \Gamma(18/5, 3/5), \tau_{Y_1} \sim \Gamma(18/5, 3/5), \tau_{Y_2} \sim \Gamma(18/5, 3/5)$, and observational noises $\sim N(0, Y_l(t) + 10)$ for $l = 1, 2$. (**c**) Using these trajectories, we estimated kinetic parameters $A_X, A_{Y_1}, A_{Y_2}, A_{Y_2 \to X}$, $K_{M_1}, K_{M_2}, K_{M_3}$, and mean delay μ_{τ_X} by assuming that B, τ_{Y_1}, and τ_{Y_2} are known. The posterior distributions of $A_{Y_1}, A_{Y_2}, K_{M_1}, K_{M_2}$, and K_{M_3} are biased, and those of $A_{Y_2 \to X}$ and K_{M_3} showed the high variances. (**d**) Such biases and variances are mainly due to the strong correlations between the pairs of parameters: (A_{Y_1}, K_{M_1}), (A_{Y_2}, K_{M_2}), and $(A_{Y_2 \to X}, K_{M_3})$. The strong correlations lead to identifiability issues between the parameters, which is also seen in the two-step activation model (Fig. 3). (**e**) Consequently, the estimate of the mean delay μ_{τ_X} was also biased. (**f**) To resolve this identifiability issue, we assumed that K_{M_1}, K_{M_2}, and K_{M_3} are known as done in the two-step activation model (Fig. 3). We then obtained accurate estimates of kinetic parameters, $A_X, A_{Y_1}, A_{Y_2}, A_{Y_2 \to X}$. (**g**) Consequently, we also obtained an accurate estimate of the mean delay, μ_{τ_X}.

References

Choi, B. *et al.* (2020). Bayesian inference of distributed time delay in transcriptional and translational regulation. *Bioinformatics*, **36**(2), 586–593.

Gillespie, D. T. (2001). Approximate accelerated stochastic simulation of chemically reacting systems. *The Journal of Chemical Physics*, **115**(4), 1716–1733.

Vihola, M. (2012). Robust adaptive metropolis algorithm with coerced acceptance rate. *Statistics and Computing*, **22**(5), 997–1008.
